# Site-Specific Perturbations of Alpha-Synuclein Fibril Structure by the Parkinson's Disease Associated Mutations A53T and E46K

**DOI:** 10.1371/journal.pone.0049750

**Published:** 2013-03-07

**Authors:** Luisel R. Lemkau, Gemma Comellas, Shin W. Lee, Lars K. Rikardsen, Wendy S. Woods, Julia M. George, Chad M. Rienstra

**Affiliations:** 1 Department of Chemistry, University of Illinois at Urbana-Champaign, Urbana, Illinois, United States of America; 2 Center for Biophysics and Computational Biology, University of Illinois at Urbana-Champaign, Urbana, Illinois, United States of America; 3 Department of Cell and Developmental Biology, University of Illinois at Urbana-Champaign, Urbana, Illinois, United States of America; 4 Department of Biochemistry, University of Illinois at Urbana-Champaign, Urbana, Illinois, United States of America; University of Padova, Italy

## Abstract

Parkinson's disease (PD) is pathologically characterized by the presence of Lewy bodies (LBs) in dopaminergic neurons of the substantia nigra. These intracellular inclusions are largely composed of misfolded α-synuclein (AS), a neuronal protein that is abundant in the vertebrate brain. Point mutations in AS are associated with rare, early-onset forms of PD, although aggregation of the wild-type (WT) protein is observed in the more common sporadic forms of the disease. Here, we employed multidimensional solid-state NMR experiments to assess A53T and E46K mutant fibrils, in comparison to our recent description of WT AS fibrils. We made *de novo* chemical shift assignments for the mutants, and used these chemical shifts to empirically determine secondary structures. We observe significant perturbations in secondary structure throughout the fibril core for the E46K fibril, while the A53T fibril exhibits more localized perturbations near the mutation site. Overall, these results demonstrate that the secondary structure of A53T has some small differences from the WT and the secondary structure of E46K has significant differences, which may alter the overall structural arrangement of the fibrils.

## Introduction

Aggregation of the neuronal protein alpha-synuclein (AS) is strongly implicated in both sporadic and familial forms of Parkinson's disease (PD), and with a variety of other neurodegenerative diseases collectively termed synucleinopathies. AS is the main protein component of Lewy bodies, intraneuronal cytoplasmic inclusions that are the defining neuropathological hallmark of PD [1]. Missense mutations in AS, including A53T [2], A30P [3], and E46K [4], are associated with early-onset disease, suggesting that the mutant proteins differ either in their aggregation propensity or in the stability or toxicity of the resultant aggregated species.

Like wild-type (WT) AS, the mutant proteins A30P, E46K and A53T are largely unstructured in solution [5], [6], [7]. However, the mutant proteins differ in their fibrillation kinetics *in vitro*; A53T and E46K fibrillize more rapidly than WT [7], [8], while A30P fibrillizes more slowly, preferentially adopting a protofibrillar intermediate state [9]. To better resolve the potential similarities and differences among fibrils comprised of mutant and WT AS, we have employed magic angle spinning (MAS) solid-state nuclear magnetic resonance (SSNMR) spectroscopy. This technique is uniquely suited to structural studies of amyloid proteins, such as fibrillar AS [10], [11], [12], [13], which are insoluble and do not form X-ray diffraction-quality crystals. Several SSNMR-derived structures or structural models of amyloid fibrils now have been deposited in the protein data bank (PDB) [14], [15], [16], [17], [18]. Recently, we used MAS SSNMR to demonstrate that the A30P mutation does not affect the secondary structure of AS fibrils when compared site-specifically with WT AS fibrils [19].

The current study focuses on the A53T and E46K mutant fibrils. While previous reports indicated significant structural differences between WT and A53T fibrils [20], those studies were complicated by batch-to-batch variability in fibril polymorphs for the WT protein [10]. We have established carefully controlled sample preparation conditions that allow reproducible measurements of AS chemical shifts [21] and have subsequently made extensive assignments of the fibril core of WT protein [12]. The same protocol for fibril preparation that we used for WT protein is also applicable to the mutant proteins [19], allowing a more direct and complete comparison of WT and mutant fibrils. Here we show that, in contrast to a prior report [10], A53T fibrils have a highly similar secondary structure to WT fibrils, with small, localized perturbations near the mutation site, as well as for some residues numbered in the range 60–70 and 80–90. E46K fibrils present larger perturbations in secondary structure, which we attribute to the large electrostatic alteration at position 46.

## Materials and Methods

### Protein sample preparation

Uniformly (U)-^15^N, ^13^C labeled WT, A53T and E46K AS were prepared following previously published protocols [21]. In summary, recombinant protein expression was performed in *E. coli* BL21(DE3) using minimal media supplemented with BioExpress (Cambridge Isotopes). Purification was performed by chemical lysis and two chromatographic steps (hydrophobic interaction and gel exclusion), resulting in ∼60 mg of pure protein from each L of culture. Sample purity was confirmed by SDS-PAGE gel electrophoresis and mass spectrometry.

### α-Synuclein fibrillation

Solutions of monomeric (WT, A53T or E46K) full-length AS (1 mM, 50 mM phosphate buffer, pH 7.5, 0.02% azide, 0.1 mM EDTA) were syringe filtered (0.22 µm) and seeded with natural abundance AS fibrils of the corresponding protein (WT, A53T or E46K). Samples were then incubated with shaking (200 rpm) at 37°C, as previously described [21].

### Electron microscopy

A53T, E46K and WT AS fibril samples were treated with Karnovsky's fixative. After negatively staining with 2% ammonium molybdate (w/v), samples were applied on formvar carbon coated grids (300 mesh). Samples were viewed with a Hitachi H600 Transmission Electron Microscope operating at 75 kV.

### α-Synuclein fibril preparation for solid-state NMR experiments

After 3 weeks of fibrillation, samples were ultracentrifuged for 1 hr at 100,000 *g.* The resultant pellets were washed with distilled water and ultracentrifuged again for 1 hr at 100,000 *g*. The supernatant was removed and the fibril pellets were dried under a stream of N_2_ (*g*) until the final mass was unchanged. The dry powders were packed into 3.2 mm (thin or standard wall) NMR rotors (Varian, Inc., Palo Alto and Walnut Creek, CA and Fort Collins, CO; now part of Agilent Technologies, Santa Clara, CA and Loveland, CO), rehydrated to 36% water by mass and kept hydrated by Kel-F and rubber spacers, as previously described [12].

### Solid-state NMR data collection and analysis

A 14.1 Tesla (600 MHz, ^1^H frequency) Agilent Infinity Plus spectrometer equipped with a 3.2 mm T3 Agilent Balun™ ^1^H-^13^C-^15^N MAS probe, was utilized to perform all E46K SSNMR experiments. A 17.6 Tesla (750 MHz, ^1^H frequency) Agilent VNMRS spectrometer equipped with a Varian 3.2 mm Balun™ ^1^H-^13^C-^15^N MAS probe, was utilized to perform all A53T SSNMR experiments. Experiments utilized tangent ramped cross polarization [22] and SPINAL-64 [23], [24] ^1^H decoupling during acquisition and evolution periods with ∼75 kHz of field strength. For 3D ^15^N-^13^C-^13^C and ^13^C-^15^N-^13^C correlation experiments, band-selective SPECIFIC cross-polarization (CP) [25] was employed for heteronuclear polarization transfer between ^15^N and ^13^C and DARR [26] mixing for ^13^C homonuclear polarization transfer. All E46K experiments were acquired under 13.3 kHz MAS and at a variable temperature (VT) of 10°C. All A53T experiments were acquired under 12.5 kHz MAS and at a VT temperature of 0°C. 1D ^13^C and 2D ^13^C-^13^C were also collected at a range of temperatures from −10°C to +20°C to confirm that the spectra did not depend to a significant extent on the exact sample temperature. Chemical shifts were externally referenced using adamantane, with the downfield ^13^C peak at 40.48 ppm [27].

NMR datasets were processed in NMRPipe [28] by applying back linear prediction to the direct dimension, and in all dimensions Lorentzian-to-Gaussian apodization was applied prior to zero filling and Fourier transformation. Peak picking, assignments and peak heights were obtained with NMRViewJ software [29] using Gaussian peak integration methods. Chemical shifts were deposited in the Biological Magnetic Resonance Bank (BMRB codes #18208 and #18207 for E46K and A53T, respectively).

## Results and Discussion

### Sample characterization

**Figure 1:**
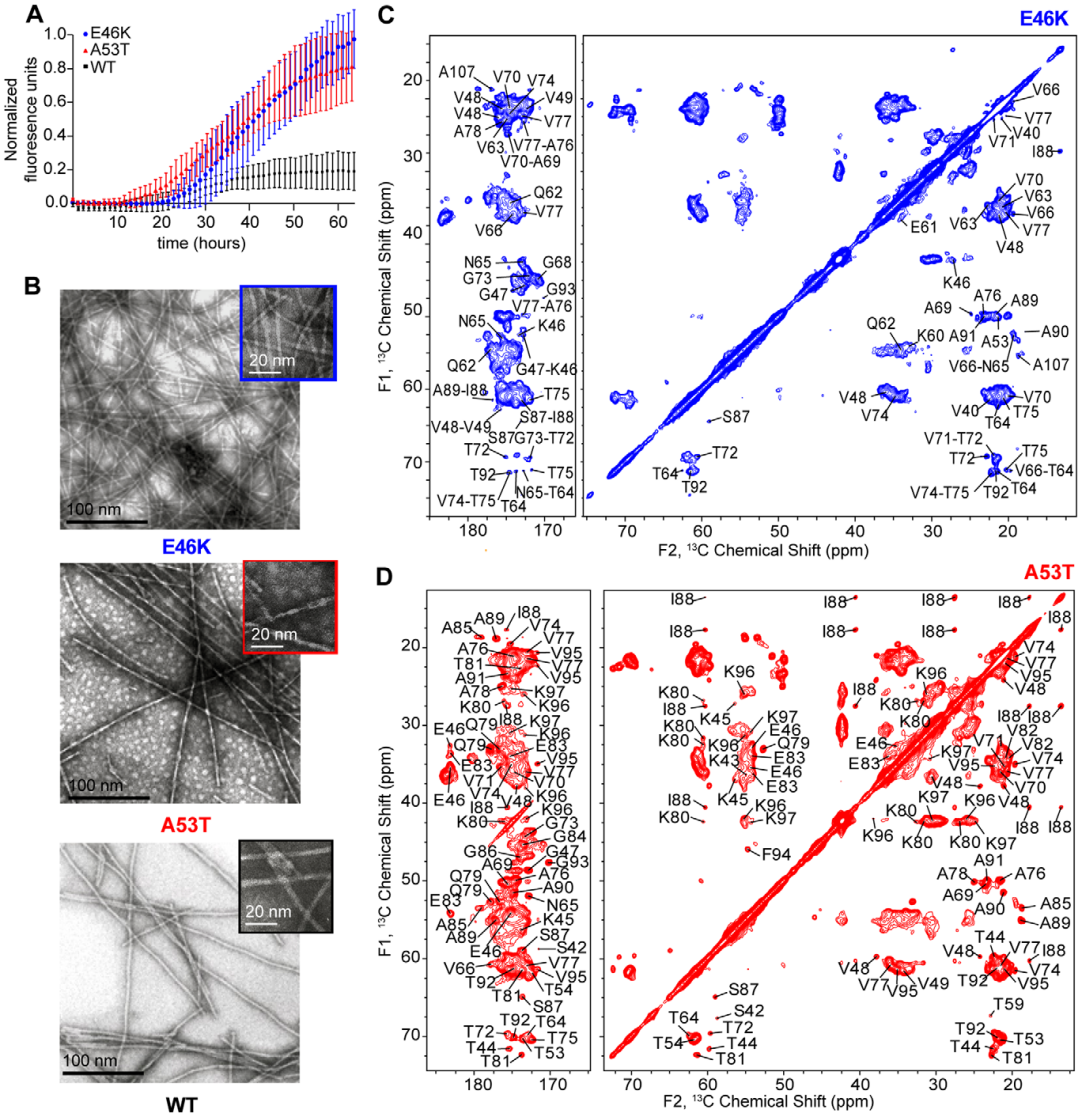
Fibrils of mutant AS proteins prepared *in vitro* have a highly homogeneity and morphology similar to WT fibrils. (A) AS fibrils formation of (blue circles) E46K, (red triangles) A53T and (black squares) WT monitored by the Thioflavin T fluorescence assay. Error bars were determined from seven replicates for each. Measurements were normalized to the highest fluorescence intensity obtained across all samples. (B) Comparison of the electron micrographs of (top) E46K, (middle) A53T and (bottom) WT AS fibrils. ^13^C-^13^C 2D with 50 ms DARR mixing of (C) E46K and (D) A53T AS fibrils.

Monomers of each AS sample were incubated at 37°C and 200 rpm shaking, and fibrillation kinetics were measured by ThT fluorescence under identical conditions to the preparation of the NMR samples. The A53T and E46K mutant proteins exhibited accelerated fibrillation kinetics relative to WT (Figure 1A), consistent with previous reports [7], [8], [9], [30], [31], [32], [33]. Previous studies indicate possible differences in morphology between the mutants and WT based on FE and AFM investigations [13], [34], while similar morphologies were observed by electron microscopy [32], [35] (Figure 1B). Under these conditions, the rehydrated SSNMR samples of both mutants yielded high resolution ^13^C-^13^C 2D spectra (Figure 1C,D), with narrow linewidths (<0.2 ppm) indicating a high degree of homogeneity throughout the fibrils, as previously observed for the WT [12]. The spectra were therefore determined to have more than sufficient sensitivity and resolution to perform chemical shift assignments in combination with 3D experiments.

### Chemical shift comparison of the A53T and E46K mutants versus wild-type

**Figure 2:**
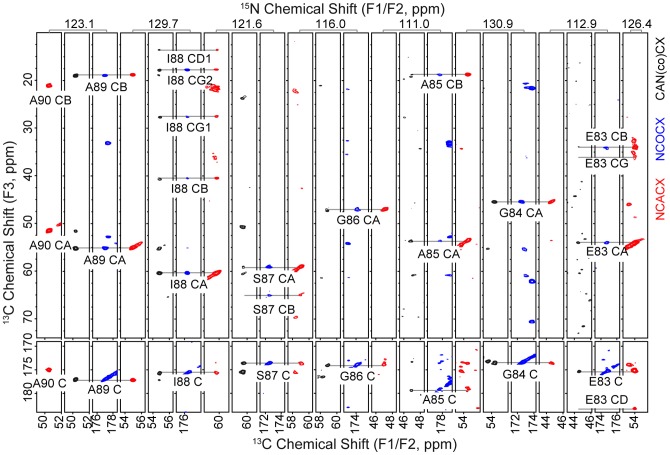
Sequential backbone-walk used to obtain the chemical shift assignments of A53T. Illustration of backbone connectivity through the NCACX (red), NCOCX (blue) and CAN(co)CX (black) spectra of residues A90-E83. In all cases the homonuclear mixing was achieved with 50 ms DARR.

Chemical shifts were assigned using the standard complement of multidimensional SSNMR experiments for both mutant fibrils, as listed in Table S1. The analysis proceeded by *de novo* backbone walks and did not rely upon the WT assignments. Common amide frequencies in the three 3D spectra—NCACX, NCOCX and CAN(co)CX—were used to establish sequential connectivities, and side-chain chemical shifts were confirmed in combination with the 2D ^13^C-^13^C spectra, resulting in site-specific assignments, as illustrated in Figure 2. Assignments were further confirmed by examining the strips through the CA frequencies in the NCACX and CAN(co)CX experiments and the carbonyl carbon (CO) frequency in the NCOCX and CAN(co)CX experiments. The residues assigned sequentially for E46K were K46-V49, T59-Q79, G84-F94 and G106-P108. The residues assigned sequentially for A53T were S42-V49, V52-V55 and T59-K97. Chemical shift assignments are included in Tables S2 and S3 for E46K and A53T, respectively.

**Figure 3:**
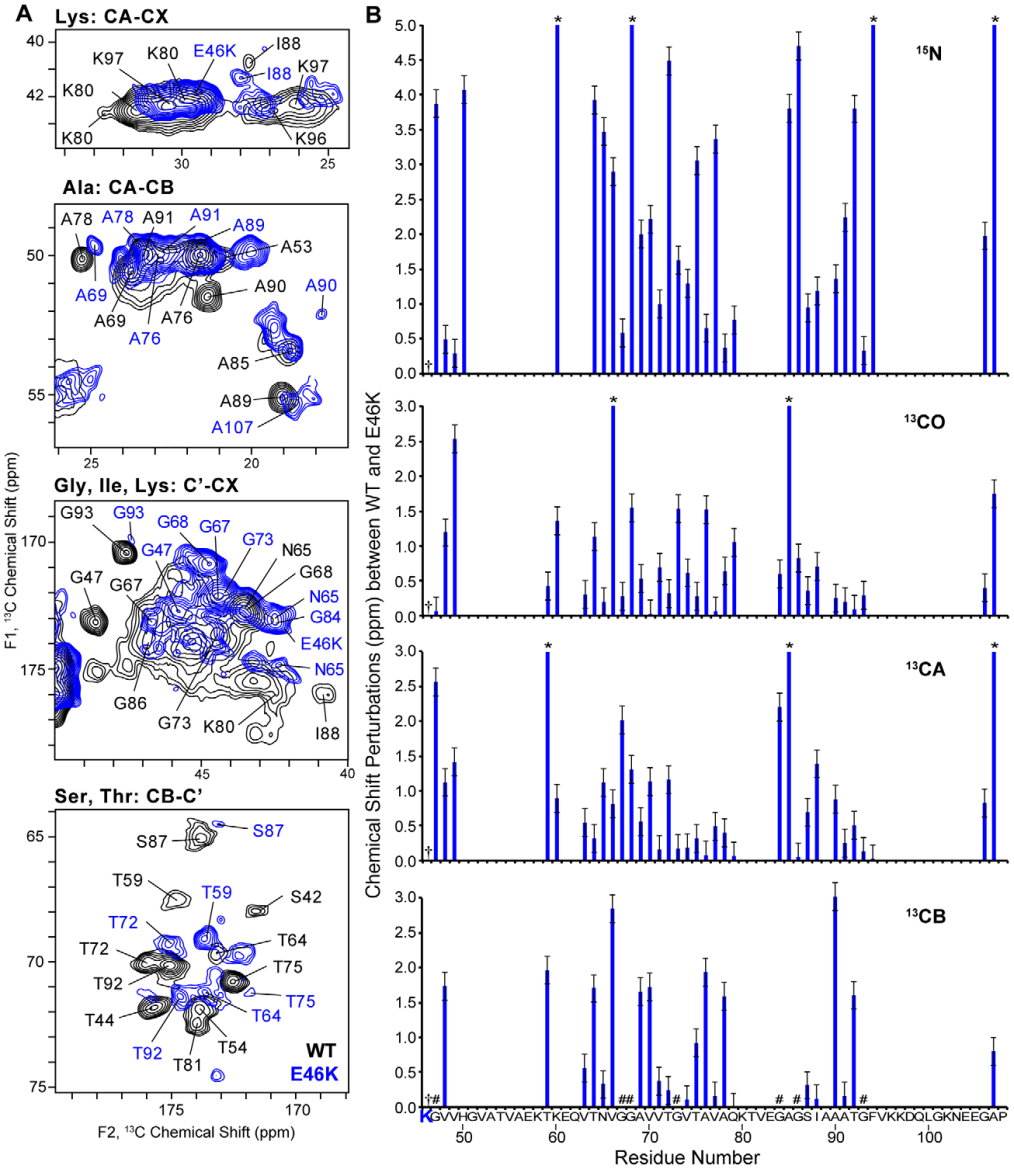
The E46K mutation causes major chemical shift perturbations throughout the AS fibril sequence. (A) Expansions of ^13^C-^13^C 2D spectral overlays (50 ms DARR mixing) of WT (black) and E46K (blue) AS fibril samples. (B) Plot of chemical shift perturbations between WT and E46K chemical shifts versus residue number. Residues labeled as (*) correspond to perturbations greater than 5 ppm (^15^N) or 3 ppm (^13^C). Residues labeled as (#) correspond to glycines. The mutation is indicated with (†). Error bars correspond to the chemical shift variations from one WT batch to another. WT chemical shift assignments were obtained from the BMRB #16939.

**Figure 4:**
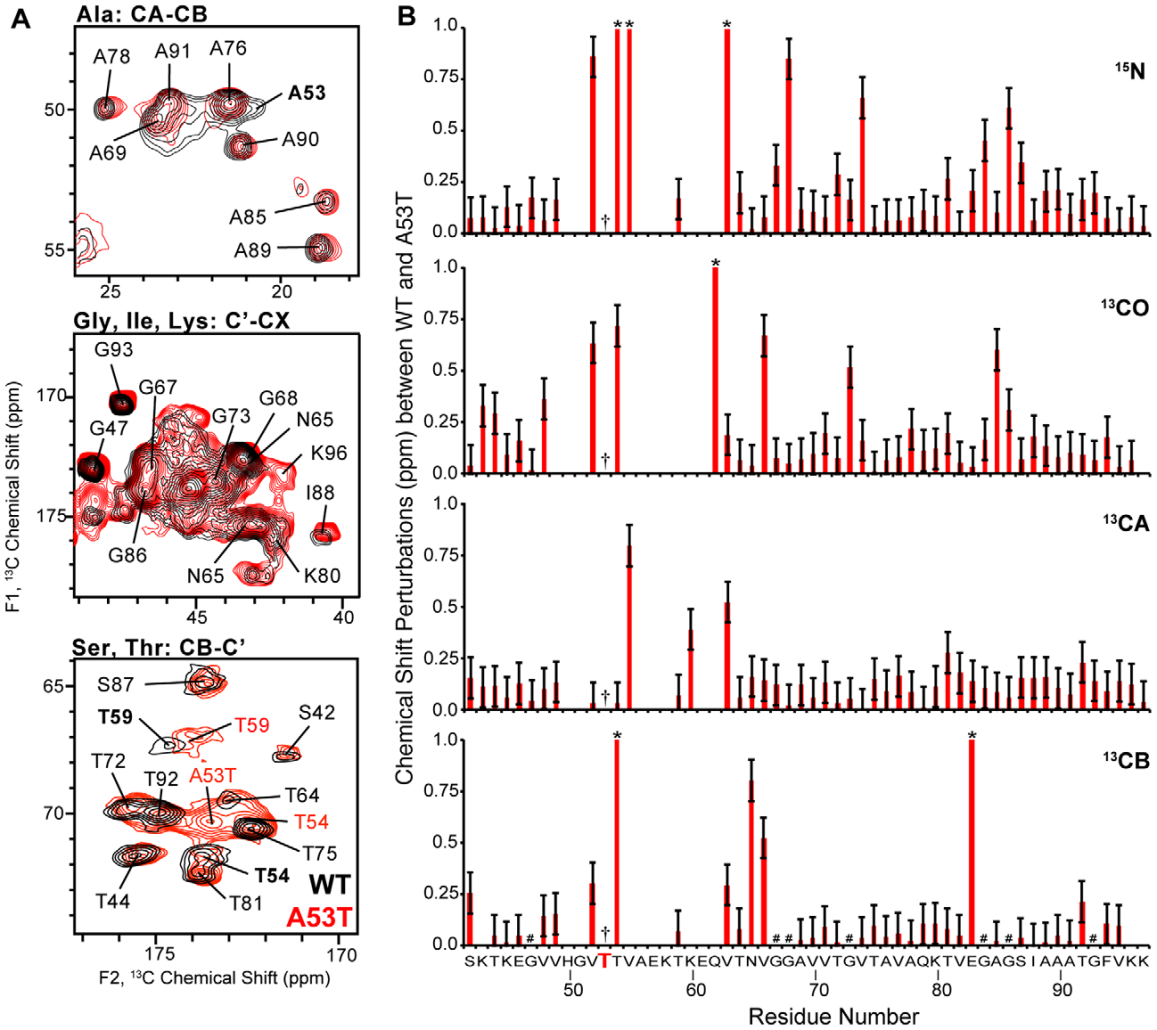
The A53T mutation causes minor perturbations throughout the AS fibril sequence. (A) Expansions of ^13^C-^13^C 2D spectral overlays (50 ms DARR mixing, 600 MHz ^1^H frequency and 13.3 kHz MAS) of WT (black) and A53T (red) AS fibril samples. (B) Plot of the chemical shift perturbations between WT and A53T chemical shifts versus residue number. Residues labeled as (*) correspond to perturbations above 1 ppm. Residues labeled as (#) correspond to glycines. The mutation is indicated with (†). Error bars correspond to the chemical shift variations from one WT batch to another. WT chemical shift assignments were obtained from the BMRB #16939.

Chemical shifts (especially for ^13^C) depend strongly upon secondary structure [36], [37], [38], [39], [40] and therefore serve as highly reliable tool for evaluating structural perturbations arising from the AS mutations. As shown in Figure 3, E46K exhibits chemical shift perturbations throughout the amino acid sequence. In the WT secondary structure [12], residue E46 is located in one of the long β-sheet strands. Therefore, it is not surprising that E46K mutation, where an ionic residue is replaced with a cationic residue at pH 7.4, leads to major chemical shift perturbations (|Δδ|_avg_∼1.6 ppm) versus the WT throughout the fibril core, due to the alternation in electrostatics. In contrast, the A53T mutant exhibits relatively minor chemical shift perturbations (|Δδ|_avg_∼0.5 ppm), and the perturbations are very much localized located in certain regions of the fibril core. The ^13^CA and ^15^N chemical shift perturbations (Figure 4) in A53T AS fibrils versus the WT are found near the mutation site, as well as in the 60 s and 80 s.

### Relative signal intensities and secondary structure comparison between the mutants and wild-type

**Figure 5:**
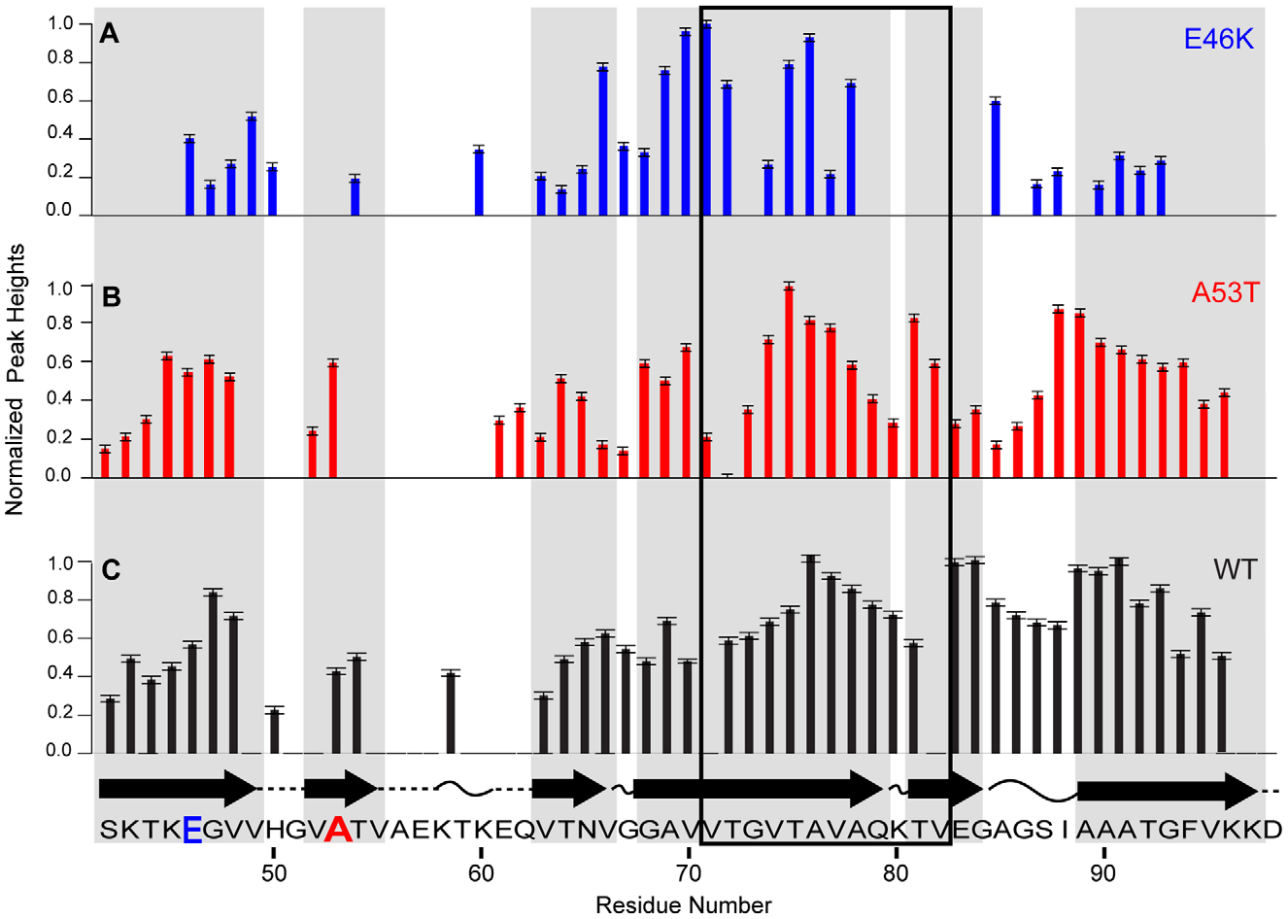
Comparison of relative signal intensities between the WT, E46K and A53T. Normalized peak heights from CANCO experiments as a function of residue number for (A) E46K, (B) A53T and (C) WT. Error bars correspond to the noise level. Representation of the secondary structure of WT AS fibrils based on TALOS+ analysis (β-strands, arrows; turn or loop curved lines; not predicted, dashed lines) from Comellas *et al*. Regions described to be essential for the fibril formation by Giasson *et al* are highlighted with a black outlined box. Grey boxes highlight the located of the WT β-strands.

The relative rigidity of regions within the fibril core was evaluated based on the signal intensities of a dipolar-based 3D CANCO experiment. Our recent investigations demonstrated that the region reported by Giasson *et al* to be responsible for fibrillation (residues 71–82 for the WT [41]) exhibited some of the highest signal intensities within the fibril core [12]. Together with several other site-specific measurements, we demonstrated that these residues are among the most rigid residues in the structure. As demonstrated in Figure 5, these regions are within the stronger regions of the core compared to the other signals in both mutants. The site-specific pattern of residues 71–82 and the relative signal intensity in the 90 s versus other regions in the sequence is somewhat different for E46K versus WT or A53T.

**Figure 6:**
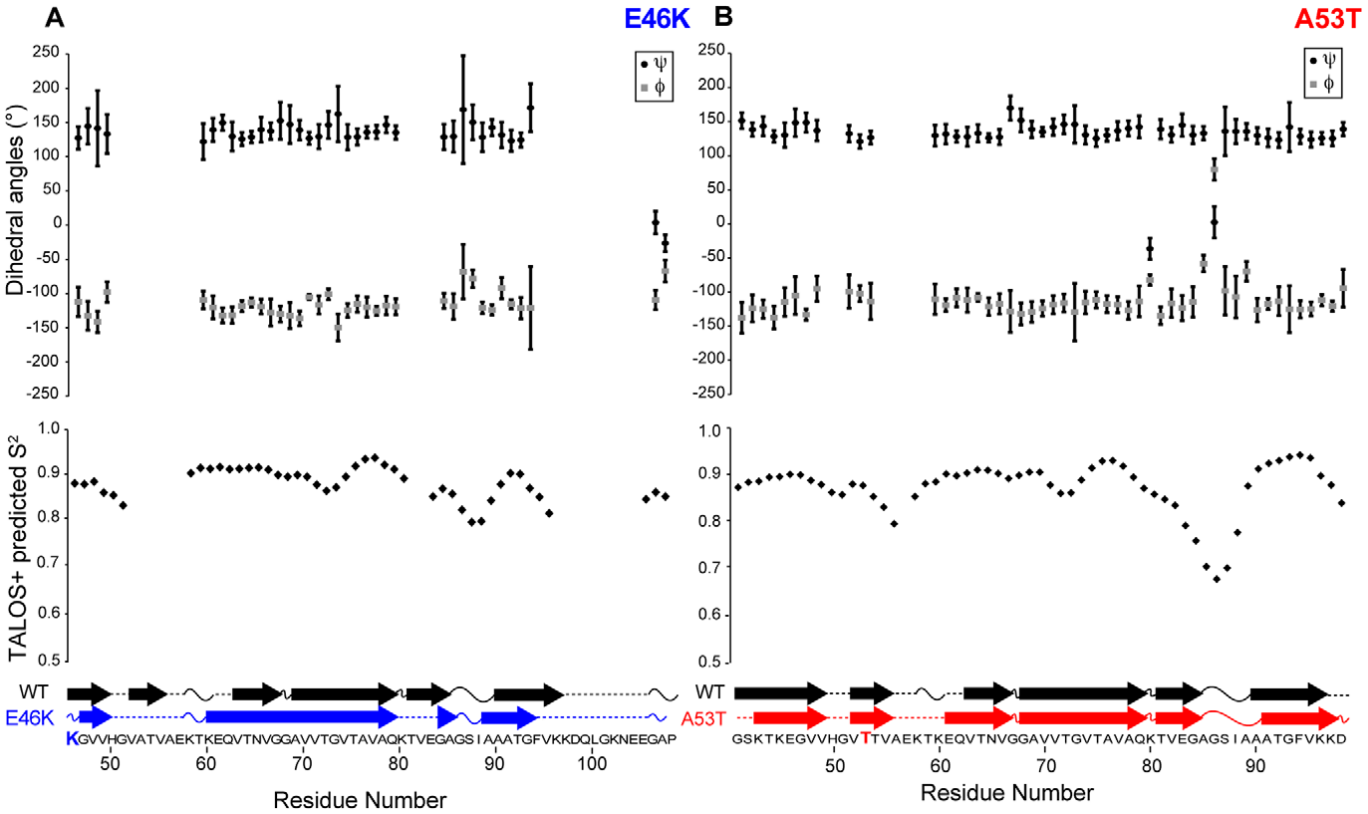
TALOS+ predicted backbone dihedral angles ψ and φ as a function of residue number. (A) E46K and (B) A53T AS fibrils. Error bars based on the 10 best TALOS+ database matches. Representation of the secondary structure for WT (black), E46K (blue) and A53T (red) AS fibrils based on TALOS+ analysis (β-strands, arrows; turn or loop curved lines; not predicted, dashed lines). WT TALOS+ results based on those from Comellas *et al*.

Because chemical shifts are highly sensitive to atomic environment [36], [37], [38], [39], [40], backbone dihedral angles (Φ and Ψ) and secondary structure can be predicted in a semi-empirical manner using the TALOS+ program [42] to obtain the backbone dihedral angles and secondary structure for E46K and A53T AS fibrils (Figure 6). While the TALOS+ results demonstrate that A53T exhibits small changes in secondary structure compared to the WT, E46K exhibits substantial chemical shift changes relative to WT, indicating differences in secondary structure patterns. These results demonstrate that the secondary structure is somewhat similar for A53T and WT; whether this arises from a common three-dimensional fold remains to be determined. In contrast, for E46K, the chemical shift values indicate significant differences in secondary structure for the E46K mutant relative to WT; it is likely that the overall structural arrangement of the E46K mutant therefore is different from that of the WT protein.

### Conclusion

In summary, we have site-specifically compared the structures of AS fibrils from the E46K and A53T early-onset PD mutants to the WT using MAS SSNMR. Our results demonstrate minor site-specific perturbations near the mutation site, the 60 s and 80 s for A53T and large chemical shift perturbations within the entire core for E46K. In addition, they reveal that the residues described to be essential for the WT fibrils formation by Giasson *et al* are also within some of the most stable residues in the core for the mutants.

Taken all these results together, this study demonstrates the presence of site-specific perturbations in the fibril structure by the A53T and E46K mutants. We believe these results will assist future investigations to elucidate how these mutants affect the fibrillogenesis of AS compared to the WT in the presence of phospholipid vesicles [43], [44]. These studies might be significantly relevant to understand the role of the early-onset PD mutants in the disease.

## Supporting Information

Table S1 **Description of the multidimensional experiments acquired to obtain** ***de novo*** **^13^C, ^15^N chemical shift assignments.**
(DOC)

Table S2 **^13^C and ^15^N chemical shift assignments of E46K AS fibrils.**
(DOC)

Table S3 **^13^C and ^15^N chemical shift assignments of A53T AS fibrils.**
(DOC)
