# Supplementary material for: Site-Specific Perturbations of Alpha-Synuclein Fibril Structure by the Parkinson's Disease Associated Mutations A53T and E46K
Source: PLoS One. 2013 Mar 7;8(3):e49750. doi: 10.1371/journal.pone.0049750 (PMC3591419; doi:10.1371/journal.pone.0049750)
Supplement: Table S1 — Description of the multidimensional experiments acquired to obtain de novo 13C, 15N chemical shift assignments. (DOC) [file pone.0049750.s001.doc]

**Table S1** Description of the multidimensional experiments acquired to obtain *de novo* ^13^C, ^15^N chemical shift assignments.

| **Mutant** | **Experiments** | **Dimensions acquired** | **Instrument**  **(^1^H frequency)** | **Mixing time and sequence** | **Acquisition Time (hrs)** | **Amount of sample (mg)*** |
| --- | --- | --- | --- | --- | --- | --- |
| E46K | CC | 2D | 600 MHz | 50 ms DARR | 9.5 | ~23 |
| E46K | N(CA)CX | 2D | 600 MHz | 50 ms DARR | 22 | ~14 |
| E46K | NCACX | 3D | 600 MHz | 50 ms DARR | 87 | ~14 |
| E46K | N(CO)CX | 2D | 600 MHz | 50 ms DARR | 2 | ~14 |
| E46K | NCOCX | 3D | 600 MHz | 50 ms DARR | 100 | ~14 |
| E46K | CA(NCO)CX | 2D | 600 MHz | 50 ms DARR | 28 | ~23 |
| E46K | CANCO | 3D | 600 MHz | - | 126 | ~23 |
| E46K | CAN(CO)CA | 3D | 600 MHz | R^2^T | 251 | ~23 |
| E46K | CAN(CO)CX | 3D | 600 MHz | 50 ms DARR | 263 | ~23 |
| A53T | CC | 2D | 600 MHz | 50 ms DARR | 9 | ~20 |
| A53T | CC | 2D | 750 MHz | 50 ms DARR | 32 | ~20 |
| A53T | N(CA)CX | 2D | 750 MHz | 50 ms DARR | 2.3 | ~20 |
| A53T | NCACX | 3D | 750 MHz | 50 ms DARR | 54 | ~20 |
| A53T | NCOCX | 3D | 750 MHz | 50 ms DARR | 40 | ~20 |
| A53T | CA(NCO)CX | 2D | 750 MHz | 50 ms DARR | 3 | ~20 |
| A53T | CANCO | 3D | 750 MHz | - | 33 | ~20 |
| A53T | CAN(CO)CX | 3D | 750 MHz | 50 ms DARR | 110 | ~20 |

*The total amount of protein was calculated based on the intensity of a ^13^C 1D DP experiment versus that of adamantane (the mass for adamantane was determined before packing the sample in the rotor).
