# Supplementary material for: Site-Specific Perturbations of Alpha-Synuclein Fibril Structure by the Parkinson's Disease Associated Mutations A53T and E46K
Source: PLoS One. 2013 Mar 7;8(3):e49750. doi: 10.1371/journal.pone.0049750 (PMC3591419; doi:10.1371/journal.pone.0049750)
Supplement: Table S2 — 13C and 15N chemical shift assignments of E46K AS fibrils. (DOC) [file pone.0049750.s002.doc]

**Table S2** ^13^C and ^15^N chemical shift assignments of E46K AS fibrils.

| **Residue** | **^15^N** | **^13^C’** | **^13^CA** | **^13^CB** | **^13^CG** | **^13^CD** | **^13^CE** |
| --- | --- | --- | --- | --- | --- | --- | --- |
| K46* | 127.8 | 173.3 | 52.5 | - | - | - | 42.2 |
| G47 | 111.8 | 172.8 | 45.8 |  |  |  |  |
| V48 | 118.1 | 175.8 | 60.6 | 36.1 | 22.6/21.4 |  |  |
| V49 | 126.4 | 172.2 | 62.7 | - | 21.4 |  |  |
| H50 | 129.9 | - | - | - | - | - | - |
| T59 | - | 173.8 | 62.0 | 69.2 | - |  |  |
| K60 | 126.1 | 174.4 | 54.0 | 33.1 | - | - | 44.0 |
| E61 | 121.0 | 174.0 | 54.9 | 34.2 | 36.8 | 179.8 |  |
| Q62 | 124.1 | 174.4 | 54.5 | 34.1 | 34.3 | 177.0 |  |
| V63 | 130.5 | 174.8 | 61.2 | 34.8 | 22.8/20.9 |  |  |
| T64 | 122.9 | 173.7 | 62.5 | 71.3 | 21.4 |  |  |
| N65 | 128.6 | 172.9 | 52.8 | 42.5 | 175.5 |  |  |
| V66 | 124.4 | 174.1 | 59.7 | 35.9 | 20.4/19.4 |  |  |
| G67 | 111.6 | 172.2 | 44.5 |  |  |  |  |
| G68 | 109.7 | 170.8 | 44.6 |  |  |  |  |
| A69 | 124.5 | 174.9 | 49.6 | 25.0 |  |  |  |
| V70 | 122.8 | 174.6 | 61.1 | 34.1 | 21.3/20.2 |  |  |
| V71 | 127.3 | 175.8 | 61.2 | 34.9 | 23.0/21.9 |  |  |
| T72 | 119.7 | 175.3 | 60.7 | 69.3 | 22.9 |  |  |
| G73 | 107.6 | 172.0 | 44.2 |  |  |  |  |
| V74 | 123.0 | 174.5 | 61.2 | 34.8 | 22.2/21.2 |  |  |
| T75 | 124.7 | 171.8 | 61.5 | 71.3 | 21.3 |  |  |
| A76 | 129.6 | 175.8 | 49.6 | 23.2 |  |  |  |
| V77 | 120.6 | 172.8 | 60.0 | 35.6 | 22.4/20.8 |  |  |
| A78 | 130.4 | 175.7 | 49.9 | 23.3 |  |  |  |
| Q79 | 119.4 | 175.5 | 52.3 | 33.0 | - | 177.6 |  |
| G84 | - | 173.0 | 42.8 |  |  |  |  |
| A85 | 127.1 | 175.2 | 50.1 | - |  |  |  |
| G86 | 116.4 | 174.4 | 46.7 |  |  |  |  |
| S87 | 116.5 | 173.1 | 59.4 | 64.5 |  |  |  |
| I88 | 122.6 | 174.9 | 61.4 | 40.3 | 27.2/17.9 | 13.3 |  |
| A89 | 127.0 | - | 50.1 | 23.3 |  |  |  |
| A90 | 124.5 | 175.0 | 52.1 | 18.0 | 17.9 |  |  |
| A91 | 125.2 | 175.8 | 49.9 | 23.3 |  |  |  |
| T92 | 121.8 | 174.8 | 61.4 | 71.4 | 21.5 |  |  |
| G93 | 115.0 | 170.3 | 47.3 |  |  |  |  |
| F94 | 114.9 | - | 54.5 | - | - | - | - |
| G106 | 104.3 | 172.2 | 43.5 |  |  |  |  |
| A107 | 117.3 | 176.9 | 55.5 | 18.8 |  |  |  |
| P108 | 118.5 | - | 61.4 | - | 27.0 | 52.8 |  |

*****Mutation site
