# Supplementary material for: Site-Specific Perturbations of Alpha-Synuclein Fibril Structure by the Parkinson's Disease Associated Mutations A53T and E46K
Source: PLoS One. 2013 Mar 7;8(3):e49750. doi: 10.1371/journal.pone.0049750 (PMC3591419; doi:10.1371/journal.pone.0049750)
Supplement: Table S3 — 13C and 15N chemical shift assignments of A53T AS fibrils. (DOC) [file pone.0049750.s003.doc]

**Table S3** ^13^C and ^15^N chemical shift assignments of A53T AS fibrils.

| **Residue** | **^15^N** | **^13^C’** | **^13^CA** | **^13^CB** | **^13^CG** | **^13^CD** | **^13^CE** | **^15^NE** |
| --- | --- | --- | --- | --- | --- | --- | --- | --- |
| S42 | 111.7 | 171.3 | 58.6 | 67.4 |  |  |  |  |
| K43 | 122.6 | 175.7 | 54.7 | 36.1 | - | - | 46.0 | - |
| T44 | 113.7 | 175.1 | 59.6 | 71.5 | 22.3 |  |  |  |
| K45 | 122.8 | 173.6 | 56.3 | 36.8 | 27.1 | - | - | - |
| E46 | 126.4 | 174.8 | 54.0 | 32.4 | 35.2 | 183.1 |  |  |
| G47 | 115.5 | 172.7 | 48.4 |  |  |  |  |  |
| V48 | 118.7 | 174.2 | 59.6 | 37.7 | 24.0/20.8 |  |  |  |
| V49 | 126.5 | - | 61.4 | 34.0 | - |  |  |  |
| V52 | - | 174.2 | 61.2 | 33.8 | 20.6 |  |  |  |
| T53* | 127.6 | 173.4 | 61.6 | 70.2 | 21.3 |  |  |  |
| T54 | 126.7 | 172.5 | 61.5 | 71.2 | 21.2 |  |  |  |
| V55 | 128.2 | - | 61.9 | - | - |  |  |  |
| T59 | 122.2 | 176.3 | 67.1 | 67.1 | 22.7 |  |  |  |
| Q62 | - | 173.7 | 54.5 | - | - |  |  | - |
| V63 | 126.3 | 174.3 | 61.2 | 33.9 | 21.2 |  |  |  |
| T64 | 126.6 | 172.8 | 62.3 | 69.5 | 21.8 |  |  |  |
| N65 | 125.4 | 172.7 | 51.7 | 43.6 | 174.9 |  |  | 113.4 |
| V66 | 127.3 | 177.9 | 60.7 | 33.6 | 21.4 |  |  |  |
| G67 | 111.5 | 172.4 | 46.8 |  |  |  |  |  |
| G68 | 103.3 | 172.5 | 43.5 |  |  |  |  |  |
| A69 | 126.6 | 175.5 | 50.5 | 23.6 |  |  |  |  |
| V70 | 120.7 | 174.5 | 60.1 | 35.8 | 21.5 |  |  |  |
| V71 | 126.4 | 176.3 | 61.1 | 35.2 | 20.9 |  |  |  |
| T72 | 115.5 | 176.3 | 59.3 | 69.5 | 22.1 |  |  |  |
| G73 | 109.4 | 173.0 | 44.1 |  |  |  |  |  |
| V74 | 123.6 | 174.9 | 61.4 | 34.9 | 21.0/19.4 |  |  |  |
| T75 | 127.8 | 172.1 | 61.9 | 70.4 | 21.4 |  |  |  |
| A76 | 130.4 | 174.3 | 49.9 | 21.3 |  |  |  |  |
| V77 | 123.9 | 173.2 | 60.7 | 35.9 | 21.3/20.4 |  |  |  |
| A78 | 130.2 | 176.1 | 49.9 | 24.9 |  |  |  |  |
| Q79 | 120.3 | 176.5 | 52.4 | 32.9 | 33.6 | 177.5 |  | 111.1 |
| K80 | 122.9 | 175.9 | 60.4 | 32.3 | 26.7 | 31.4 | 42.2 | - |
| T81 | 113.5 | 173.5 | 61.2 | 72.2 | 22.5 |  |  |  |
| V82 | 126.2 | 174.6 | 61.6 | 34.0 | 20.3 |  |  |  |
| E83 | 126.2 | 175.1 | 53.8 | 33.7 | 36.0 | 183.0 |  |  |
| G84 | 112.8 | 173.4 | 45.1 |  |  |  |  |  |
| A85 | 130.8 | 178.8 | 53.3 | 18.5 |  |  |  |  |
| G86 | 110.8 | 173.9 | 46.6 |  |  |  |  |  |
| S87 | 115.9 | 173.4 | 58.9 | 64.8 |  |  |  |  |
| I88 | 121.5 | 175.4 | 60.2 | 40.4 | 27.4/17.5 | 13.4 |  |  |
| A89 | 129.5 | 176.9 | 54.9 | 18.7 |  |  |  |  |
| A90 | 122.9 | 174.6 | 51.3 | 21.0 |  |  |  |  |
| A91 | 127.5 | 175.5 | 49.7 | 23.1 |  |  |  |  |
| T92 | 125.5 | 174.6 | 61.1 | 70.1 | 21.8 |  |  |  |
| G93 | 114.9 | 170.1 | 47.6 |  |  |  |  |  |
| F94 | 126.5 | 173.6 | 54.6 | 45.8 | - | - | - |  |
| V95 | 127.9 | 171.4 | 61.3 | 34.9 | 22.2/20.6 |  |  |  |
| K96 | 133.1 | 173.1 | 55.0 | 38.1 | 25.7 | 31.1 | 41.8 | - |
| K97 | 130.3 | 174.9 | 54.1 | 34.1 | 25.1 | 30.6 | 42.1 | - |
| D98 | 124.0 | 175.2 | 54.9 | 42.5 |  |  |  |  |
| T | 122.1 | 174.1 | 66.8 | 67.9 | 25.7 |  |  |  |

*****Mutation site
